# Supplementary material for: Genome Wide Analysis of Family-1 UDP Glycosyltransferases in Populus trichocarpa Specifies Abiotic Stress Responsive Glycosylation Mechanisms
Source: Genes (Basel). 2022 Sep 13;13(9):1640. doi: 10.3390/genes13091640 (PMC9498546; doi:10.3390/genes13091640)
Supplement: Supplementary file 1 [file genes-13-01640-s001.zip › Supplementary Figures.pdf]

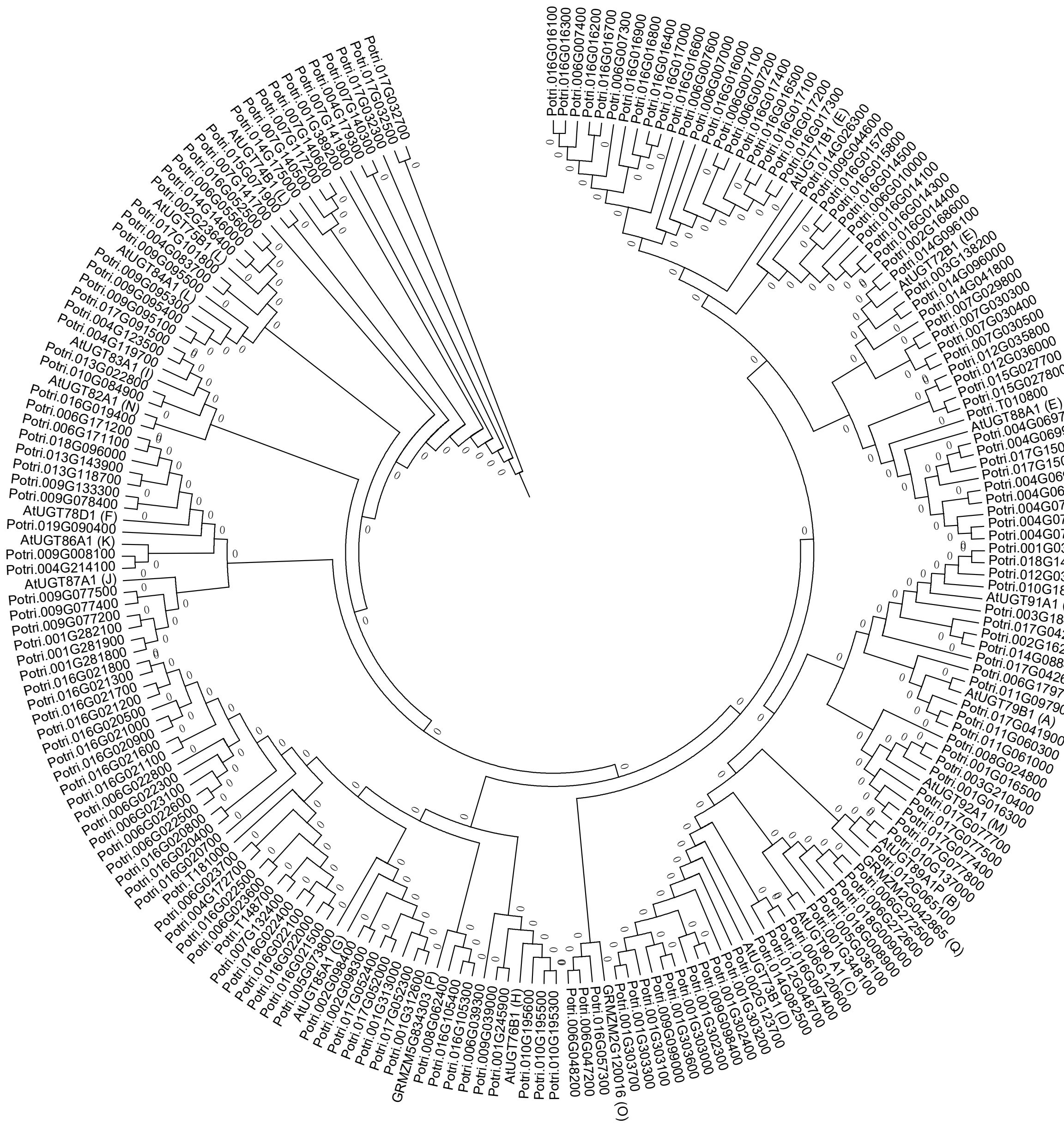

Supplementary Figure S1. Gene duplication tree and evolutionary relationships of 191 UGTs . The evolutionary history shown is from a user-provided tree. Gene duplications are identified by searching for all branching points in the topology with at least one species that is present in both subtrees of the branching point. An unrooted gene tree was used for the analysis such that the search for duplication events was performed by finding the placement of the root on a branch or branches that produced the minimum number of duplication events. There are 190 gene duplications (closed egg shaped) identified in the tree. Evolutionary analyses were conducted in MEGA7

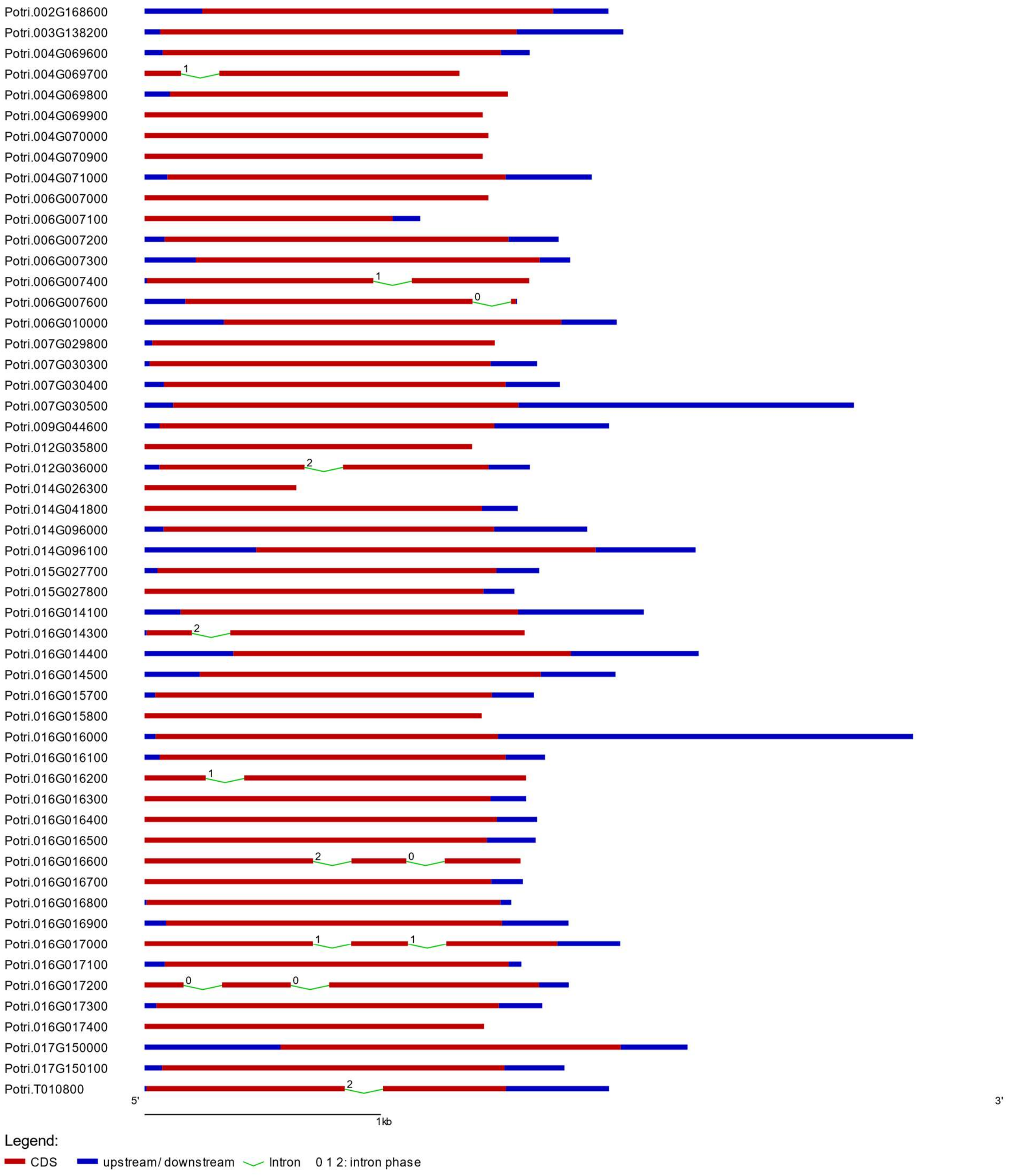
Potri.002G168600
Potri.003G138200
Potri.004G069600
Potri.004G069700
Potri.004G069800
Potri.004G069900
Potri.004G070000
Potri.004G070900
Potri.004G071000
Potri.006G007000
Potri.006G007100
Potri.006G007200
Potri.006G007300
Potri.006G007400
Potri.006G007600
Potri.006G010000
Potri.007G029800
Potri.007G030300
Potri.007G030400
Potri.007G030500
Potri.009G044600
Potri.012G035800
Potri.012G036000
Potri.014G026300
Potri.014G041800
Potri.014G096000
Potri.014G096100
Potri.015G027700
Potri.015G027800
Potri.016G014100
Potri.016G014300
Potri.016G014400
Potri.016G014500
Potri.016G015700
Potri.016G015800
Potri.016G016000
Potri.016G016100
Potri.016G016200
Potri.016G016300
Potri.016G016400
Potri.016G016500
Potri.016G016600
Potri.016G016700
Potri.016G016800
Potri.016G016900
Potri.016G017000
Potri.016G017100
Potri.016G017200
Potri.016G017300
Potri.016G017400
Potri.017G150000
Potri.017G150100
Potri.T010800
5'
3'
1kb
Legend:
CDS
upstream/ downstream
Intron
0 1 2: intron phase

E group

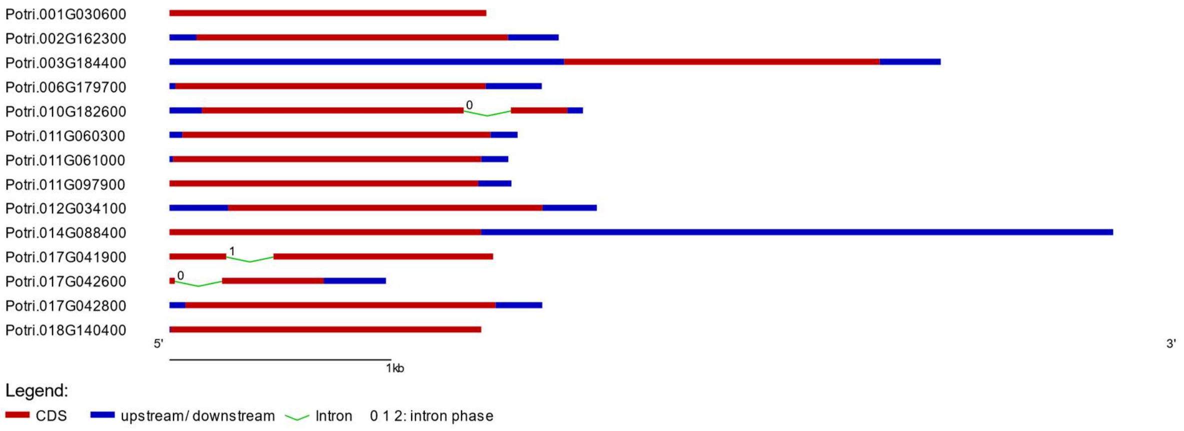

A group

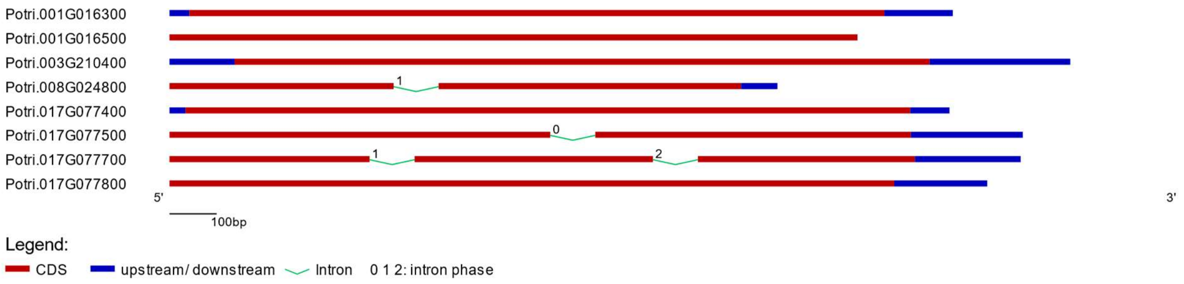

M group

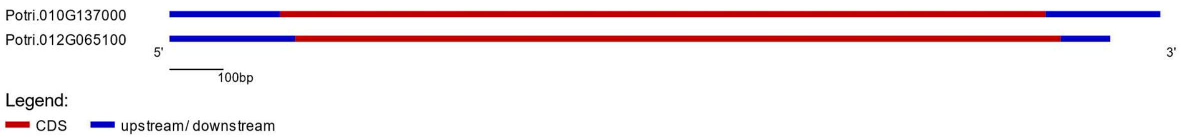

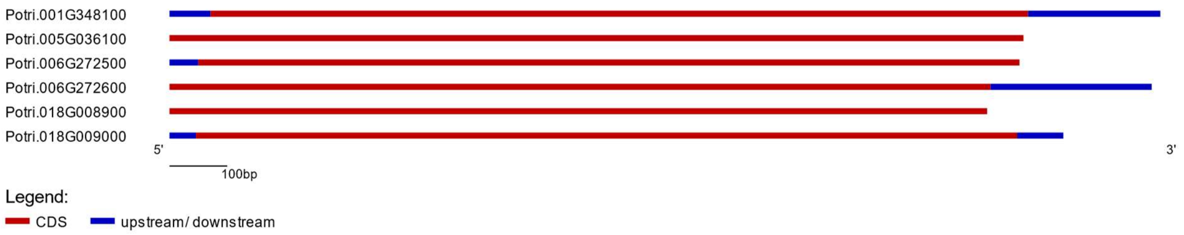

C group

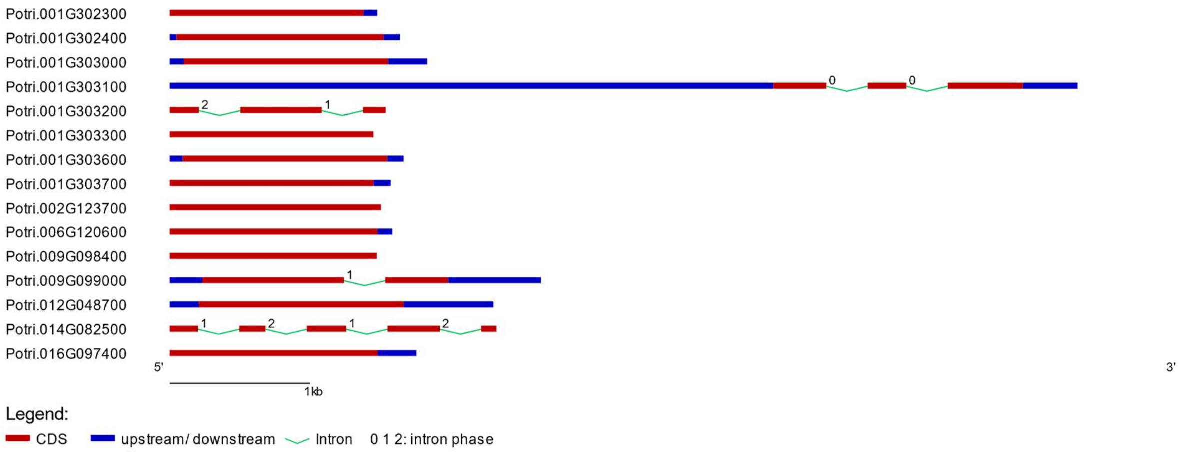

D group

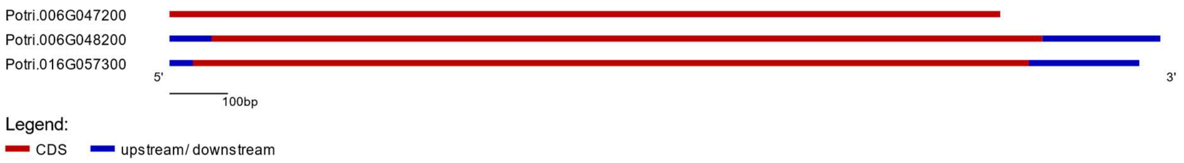

O group

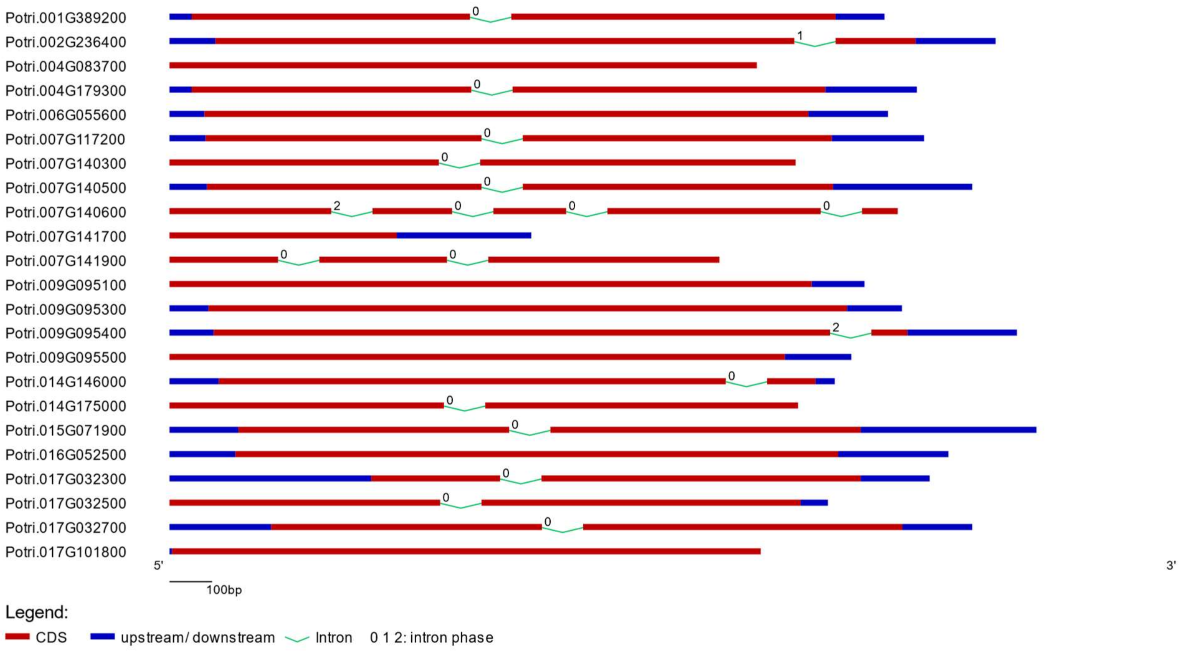

L group

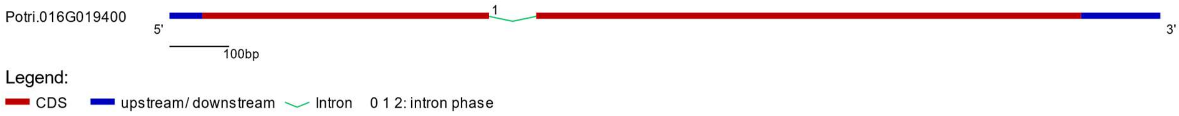

N group

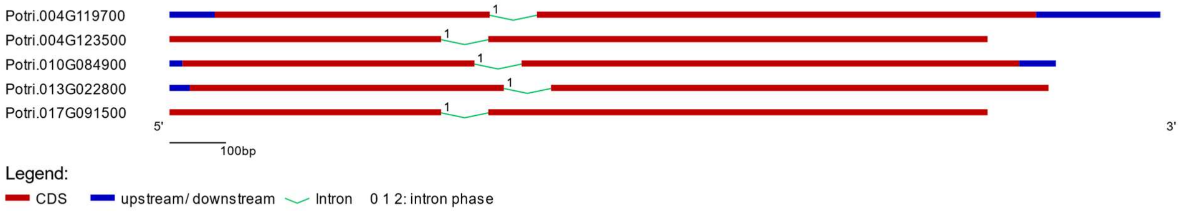

I group

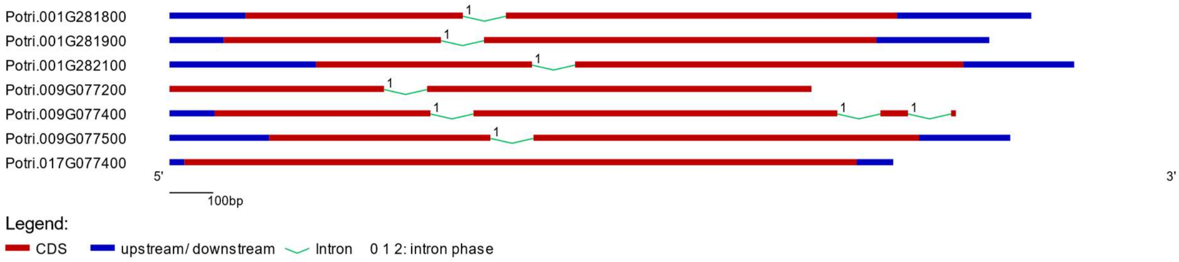

J group

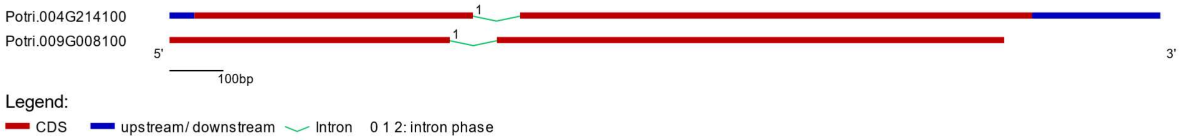

K group

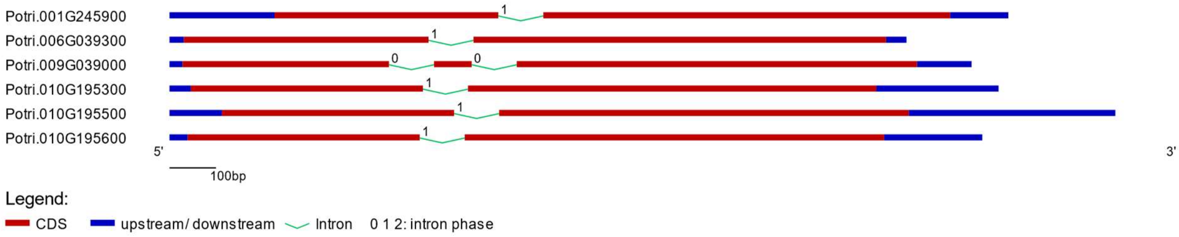

H group

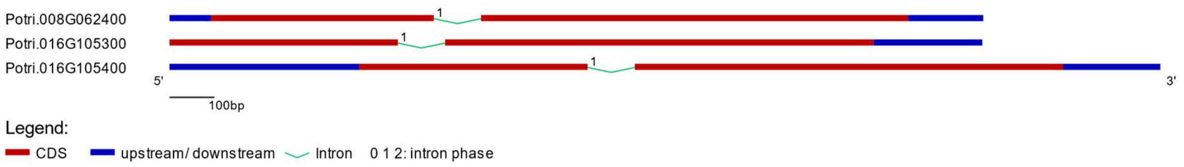

P group

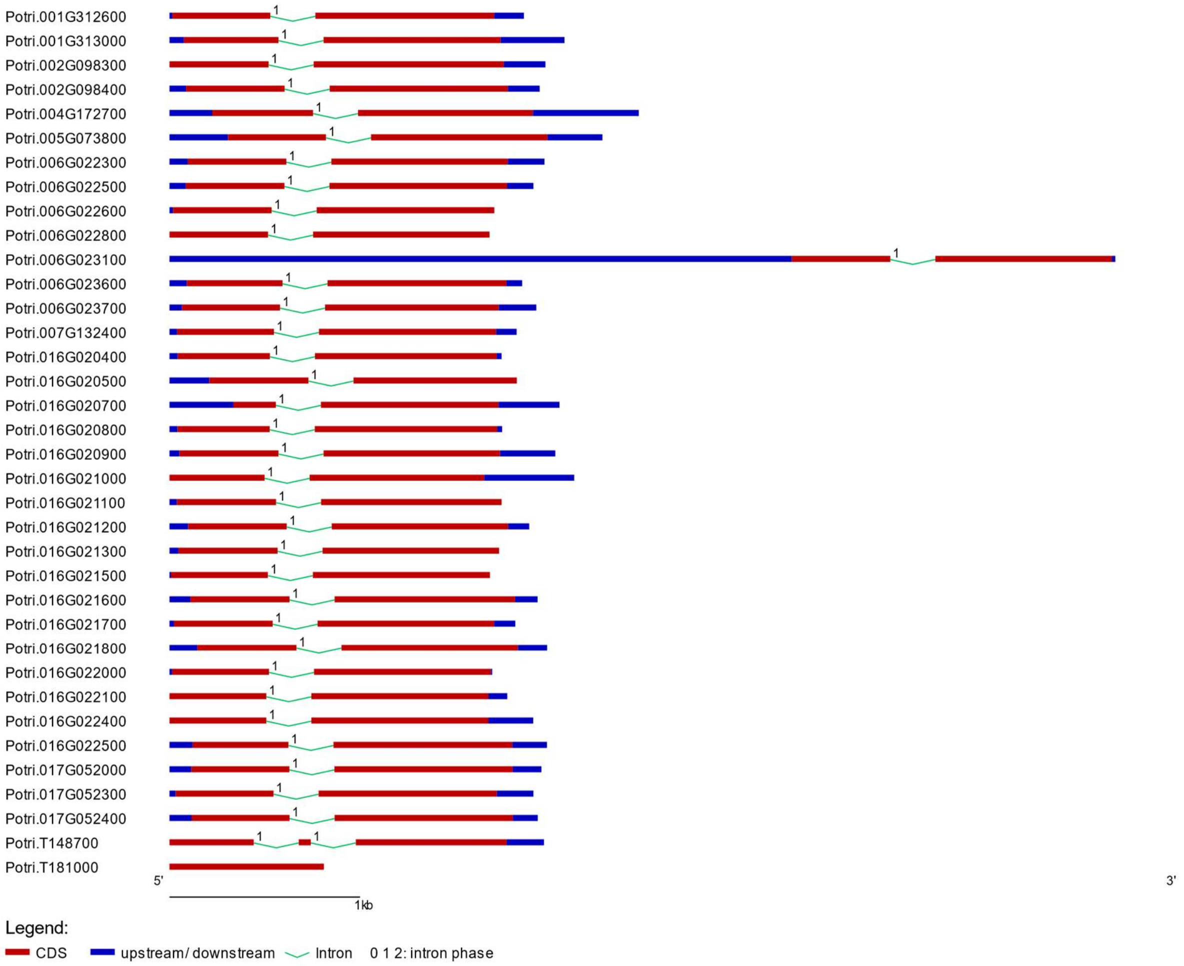

G group

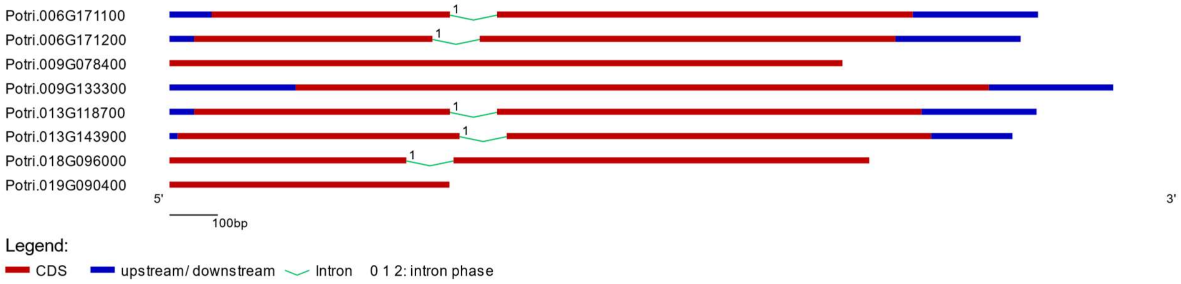

F group

Supplementary Figure S2. Gene structure of all 191 UGTs in Populus against their phylogenetic groups. Red, blue and green colors are showing the CDS, UTRs and intron regions among all the putative UGTs.

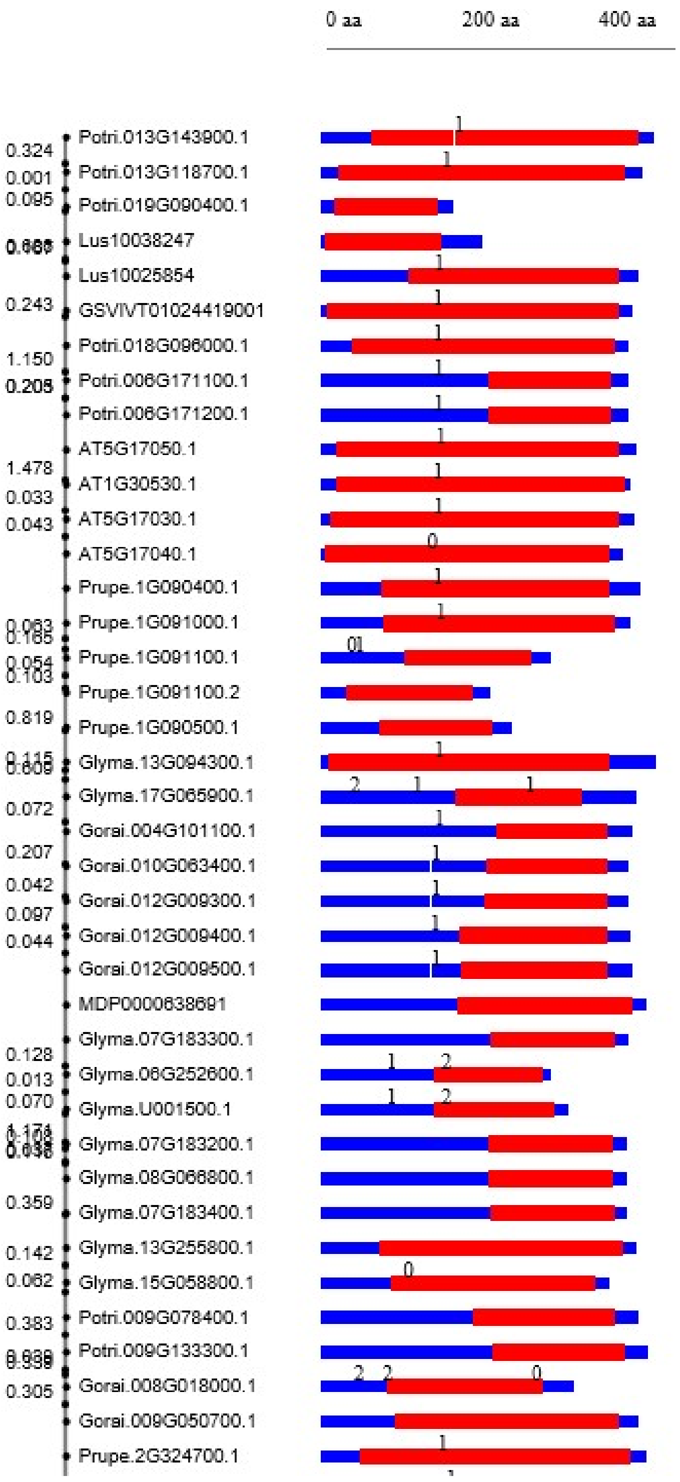

0 aa
200 aa
400 aa
Potri.013G143900.1
Potri.013G118700.1
Potri.019G090400.1
Lus10038247
Lus10025854
GSVIVT01024419001
Potri.018G096000.1
Potri.006G171100.1
Potri.006G171200.1
AT5G17050.1
AT1G30530.1
AT5G17030.1
AT5G17040.1
Prupe.1G090400.1
Prupe.1G091000.1
Prupe.1G091100.1
Prupe.1G091100.2
Prupe.1G090500.1
Glyma.13G094300.1
Glyma.17G065900.1
Gorai.004G101100.1
Gorai.010G063400.1
Gorai.012G009300.1
Gorai.012G009400.1
Gorai.012G009500.1
MDP0000638691
Glyma.07G183300.1
Glyma.06G252600.1
Glyma.U001500.1
Glyma.07G183200.1
Glyma.08G066800.1
Glyma.07G183400.1
Glyma.13G255800.1
Glyma.15G058800.1
Potri.009G078400.1
Potri.009G133300.1
Gorai.008G018000.1
Gorai.009G050700.1
Prupe.2G324700.1
0.324
0.001
0.095
0.243
1.150
1.478
0.033
0.043
0.819
0.072
0.207
0.042
0.097
0.044
0.128
0.013
0.070
0.359
0.142
0.062
0.383
0.305

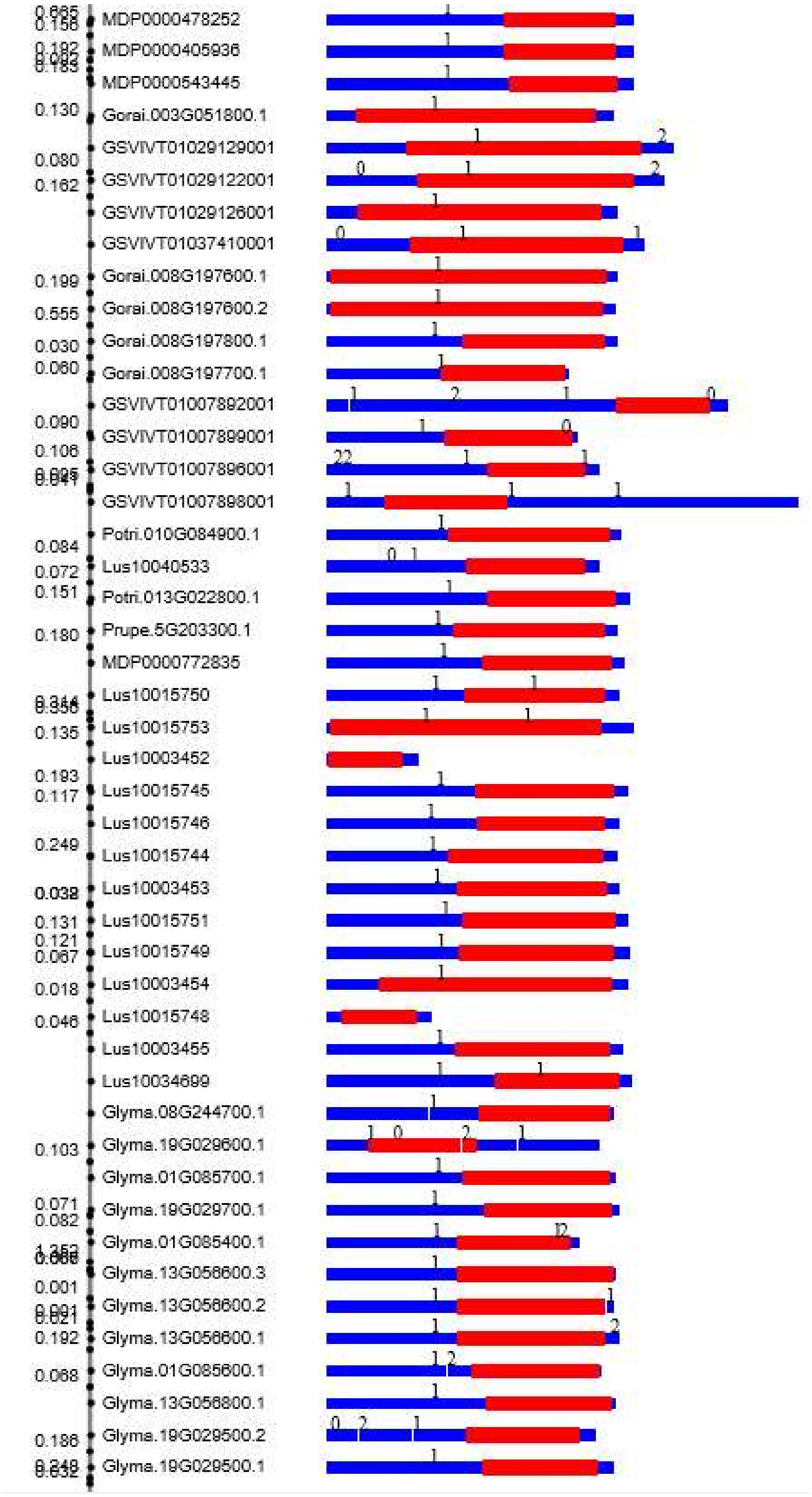
MDP0000478252
MDP0000405936
MDP0000543445
Gorai.003G051800.1
GSVIVT01029129001
GSVIVT01029122001
GSVIVT01029126001
GSVIVT01037410001
Gorai.008G197600.1
Gorai.008G197600.2
Gorai.008G197800.1
Gorai.008G197700.1
GSVIVT01007892001
GSVIVT01007899001
GSVIVT01007896001
GSVIVT01007898001
Potri.010G084900.1
Lus10040533
Potri.013G022800.1
Prupe.5G203300.1
MDP0000772835
Lus10015750
Lus10015753
Lus10003452
Lus10015745
Lus10015746
Lus10015744
Lus10003453
Lus10015751
Lus10015749
Lus10003454
Lus10015748
Lus10003455
Lus10034699
Glyma.08G244700.1
Glyma.19G029600.1
Glyma.01G085700.1
Glyma.19G029700.1
Glyma.01G085400.1
Glyma.13G056600.3
Glyma.13G056600.2
Glyma.13G056600.1
Glyma.01G085600.1
Glyma.13G056800.1
Glyma.19G029500.2
Glyma.19G029500.1
0.130
0.080
0.162
0.199
0.555
0.030
0.060
0.090
0.106
0.084
0.072
0.151
0.180
0.135
0.193
0.117
0.249
0.131
0.121
0.067
0.018
0.046
0.103
0.071
0.082
0.001
0.192
0.068
0.186

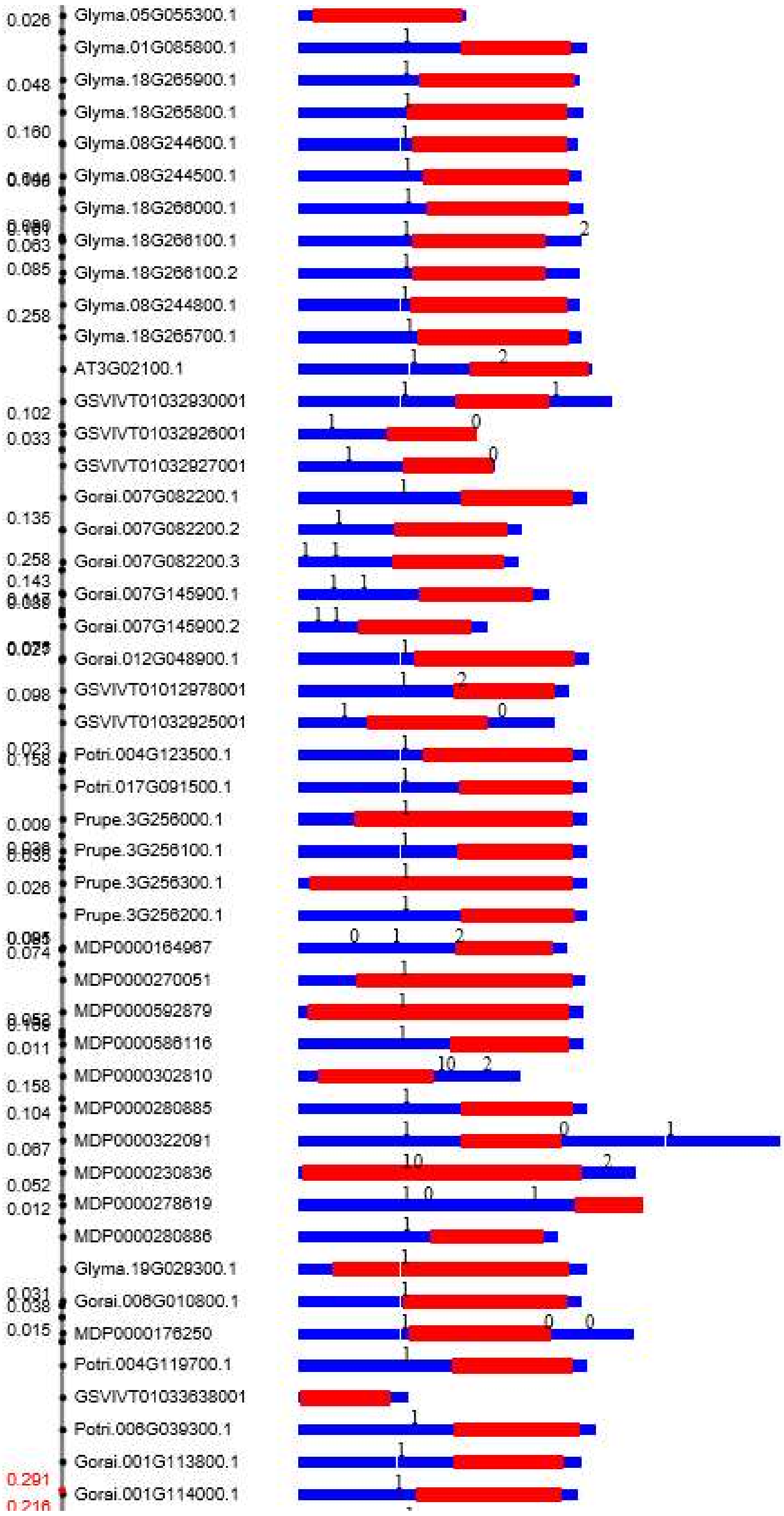

Glyma.05G055300.1
Glyma.01G085800.1
Glyma.18G265900.1
Glyma.18G265800.1
Glyma.08G244600.1
Glyma.08G244500.1
Glyma.18G266000.1
Glyma.18G266100.1
Glyma.18G266100.2
Glyma.08G244800.1
Glyma.18G265700.1
AT3G02100.1
GSVIVT01032930001
GSVIVT01032926001
GSVIVT01032927001
Gorai.007G082200.1
Gorai.007G082200.2
Gorai.007G082200.3
Gorai.007G145900.1
Gorai.007G145900.2
Gorai.012G048900.1
GSVIVT01012978001
GSVIVT01032925001
Potri.004G123500.1
Potri.017G091500.1
Prupe.3G256000.1
Prupe.3G256100.1
Prupe.3G256300.1
Prupe.3G256200.1
MDP0000164967
MDP0000270051
MDP0000592879
MDP0000586116
MDP0000302810
MDP0000280885
MDP0000322091
MDP0000230836
MDP0000278619
MDP0000280886
Glyma.19G029300.1
Gorai.006G010800.1
MDP0000176250
Potri.004G119700.1
GSVIVT01033638001
Potri.006G039300.1
Gorai.001G113800.1
Gorai.001G114000.1

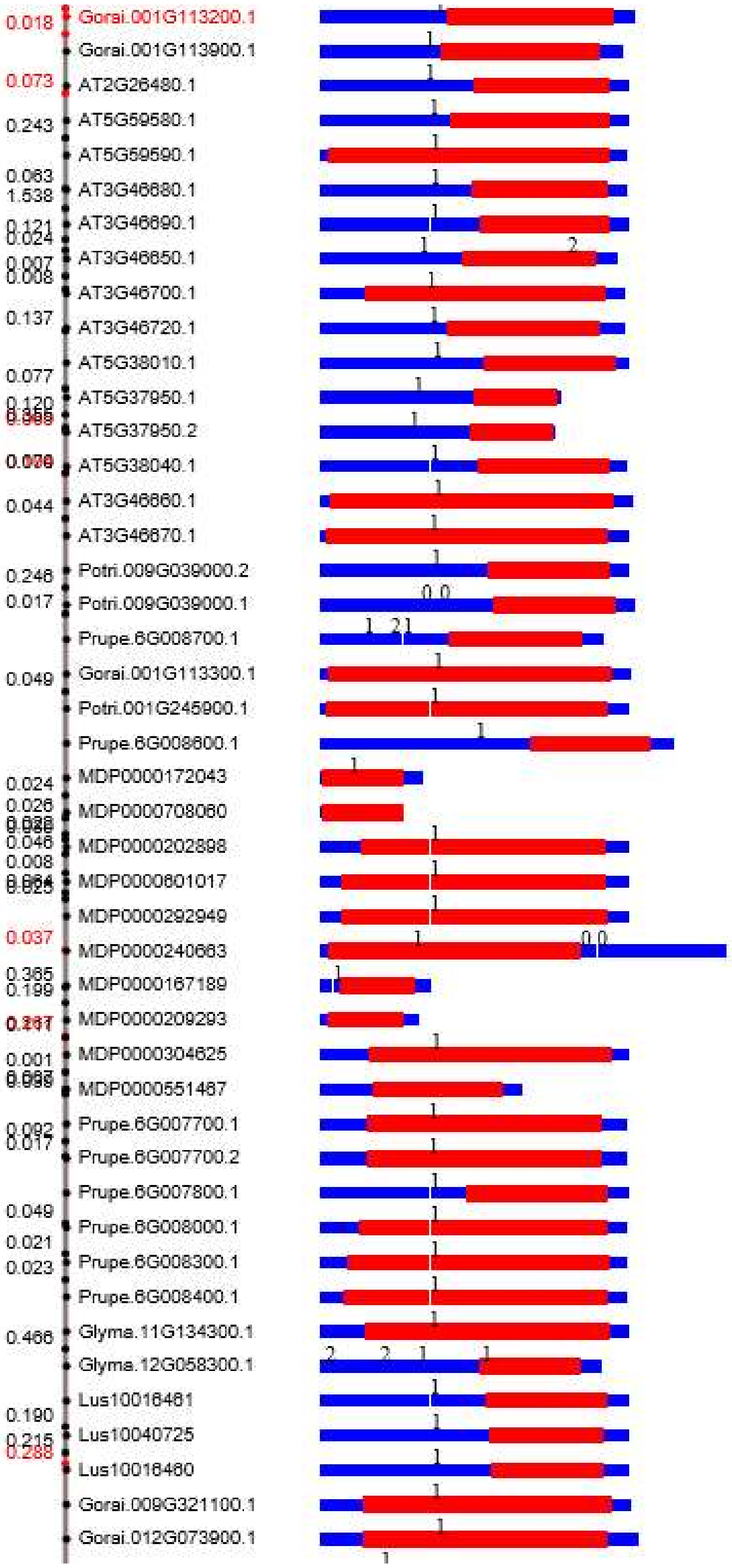

Gorai.001G113200.1
Gorai.001G113900.1
AT2G26480.1
AT5G59580.1
AT5G59590.1
AT3G46680.1
AT3G46690.1
AT3G46650.1
AT3G46700.1
AT3G46720.1
AT5G38010.1
AT5G37950.1
AT5G37950.2
AT5G38040.1
AT3G46660.1
AT3G46670.1
Potri.009G039000.2
Potri.009G039000.1
Prupe.6G008700.1
Gorai.001G113300.1
Potri.001G245900.1
Prupe.6G008600.1
MDP0000172043
MDP0000708060
MDP0000202898
MDP0000601017
MDP0000292949
MDP0000240663
MDP0000167189
MDP0000209293
MDP0000304625
MDP0000551467
Prupe.6G007700.1
Prupe.6G007700.2
Prupe.6G007800.1
Prupe.6G008000.1
Prupe.6G008300.1
Prupe.6G008400.1
Glyma.11G134300.1
Glyma.12G058300.1
Lus10016461
Lus10040725
Lus10016460
Gorai.009G321100.1
Gorai.012G073900.1

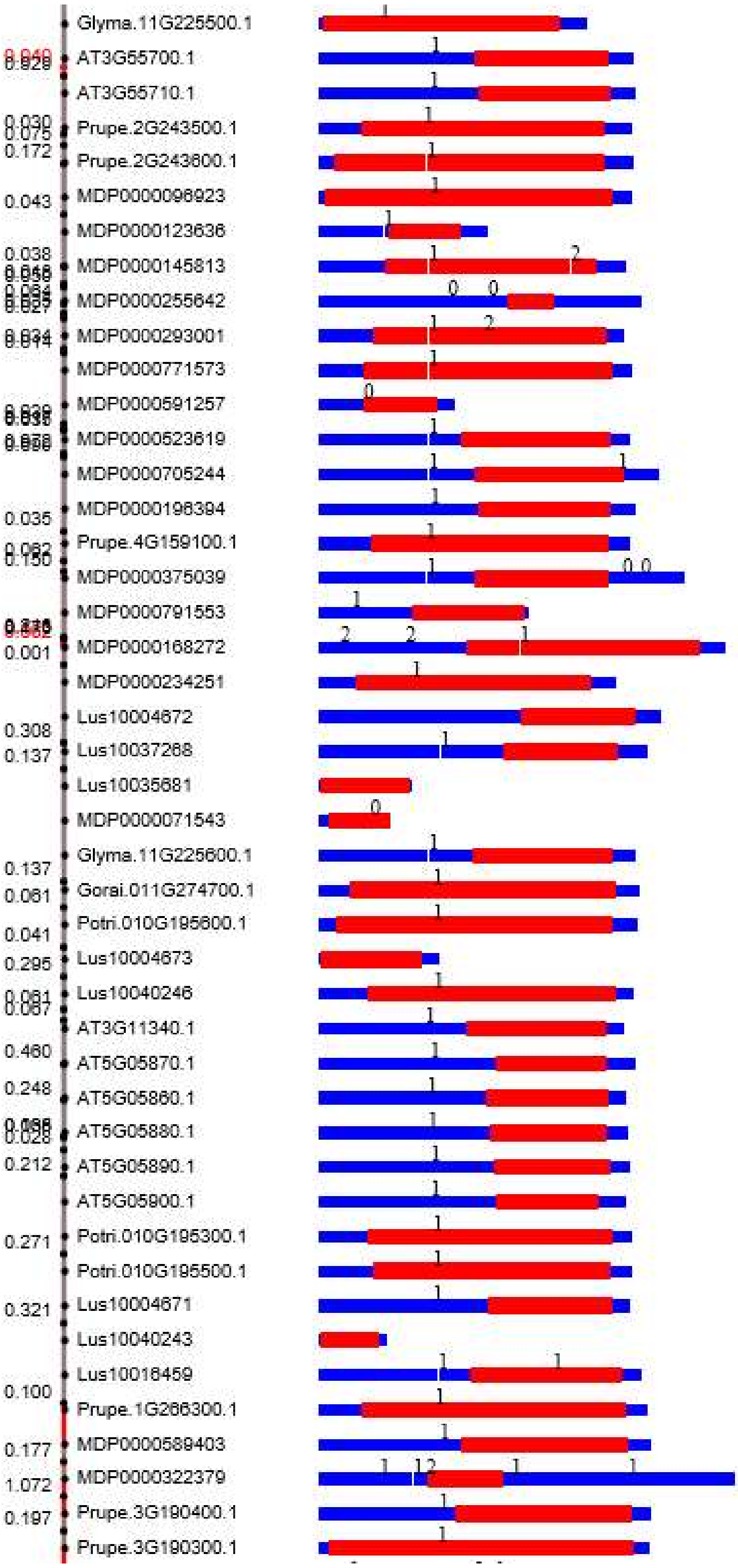
Glyma.11G225500.1
AT3G55700.1
AT3G55710.1
Prupe.2G243500.1
Prupe.2G243600.1
MDP0000096923
MDP0000123636
MDP0000145813
MDP0000255642
MDP0000293001
MDP0000771573
MDP0000591257
MDP0000523619
MDP0000705244
MDP0000196394
Prupe.4G159100.1
MDP0000375039
MDP0000791553
MDP0000168272
MDP0000234251
Lus10004672
Lus10037268
Lus10035681
MDP0000071543
Glyma.11G225600.1
Gorai.011G274700.1
Potri.010G195600.1
Lus10004673
Lus10040246
AT3G11340.1
AT5G05870.1
AT5G05860.1
AT5G05880.1
AT5G05890.1
AT5G05900.1
Potri.010G195300.1
Potri.010G195500.1
Lus10004671
Lus10040243
Lus10016459
Prupe.1G266300.1
MDP0000589403
MDP0000322379
Prupe.3G190400.1
Prupe.3G190300.1
0.030
0.075
0.172
0.043
0.038
0.035
0.062
0.150
0.001
0.308
0.137
0.137
0.061
0.041
0.295
0.061
0.067
0.460
0.248
0.028
0.212
0.271
0.321
0.100
0.177
1.072
0.197

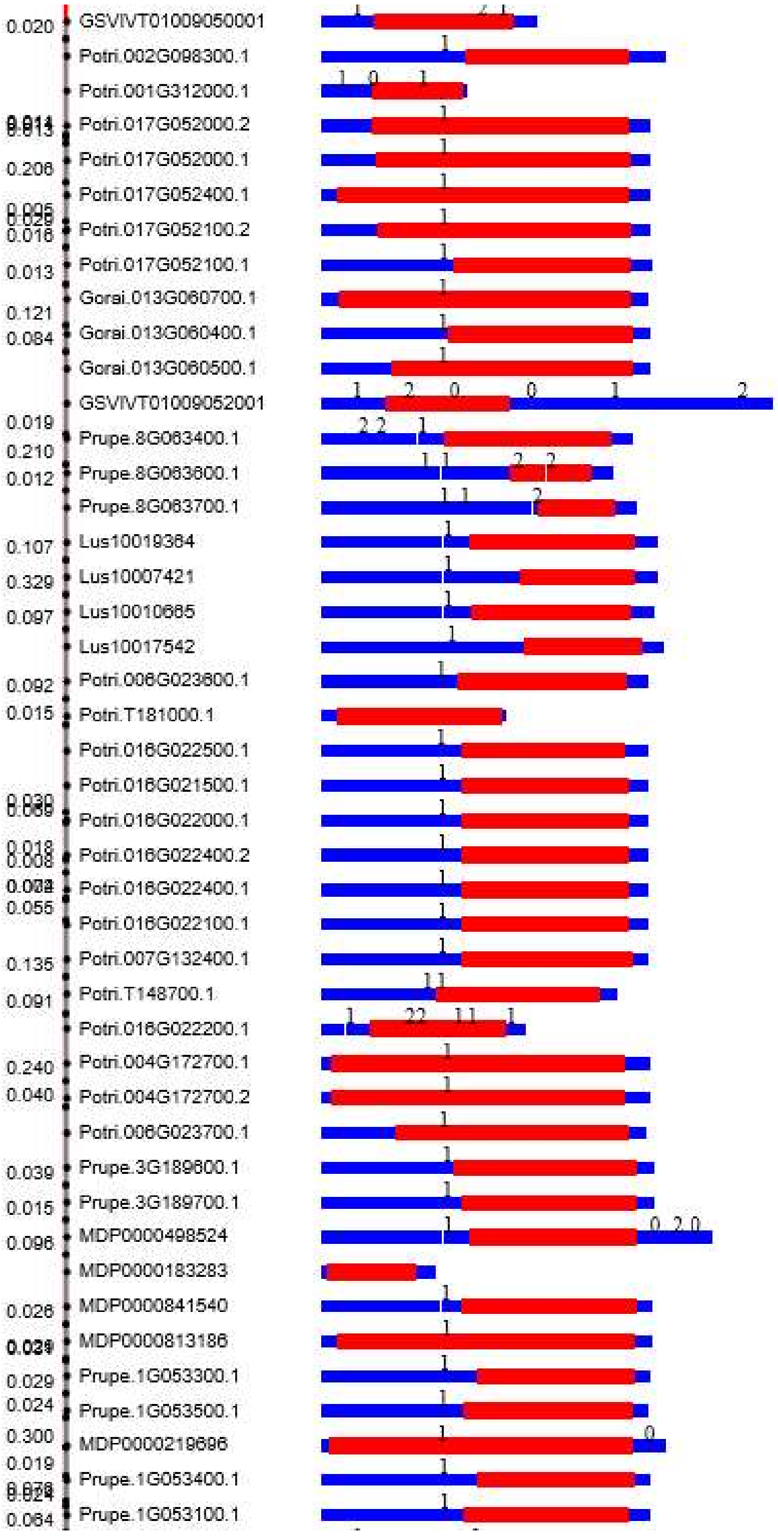

0.020
GSVIVT01009050001
Potri.002G098300.1
Potri.001G312000.1
Potri.017G052000.2
0.206
Potri.017G052000.1
Potri.017G052400.1
0.016
Potri.017G052100.2
0.013
Potri.017G052100.1
Gorai.013G060700.1
0.121
0.084
Gorai.013G060400.1
Gorai.013G060500.1
GSVIVT01009052001
0.019
0.210
Prupe.8G063400.1
0.012
Prupe.8G063600.1
Prupe.8G063700.1
0.107
Lus10019384
0.329
Lus10007421
0.097
Lus10010665
Lus10017542
0.092
Potri.006G023600.1
0.015
Potri.T181000.1
Potri.016G022500.1
Potri.016G021500.1
Potri.016G022000.1
Potri.016G022400.2
Potri.016G022400.1
0.055
Potri.016G022100.1
0.135
Potri.007G132400.1
0.091
Potri.T148700.1
Potri.016G022200.1
0.240
Potri.004G172700.1
0.040
Potri.004G172700.2
Potri.006G023700.1
0.039
Prupe.3G189600.1
0.015
Prupe.3G189700.1
0.096
MDP0000498524
MDP0000183283
0.026
MDP0000841540
MDP0000813186
0.029
Prupe.1G053300.1
0.024
Prupe.1G053500.1
0.300
MDP0000219696
0.019
Prupe.1G053400.1
0.064
Prupe.1G053100.1

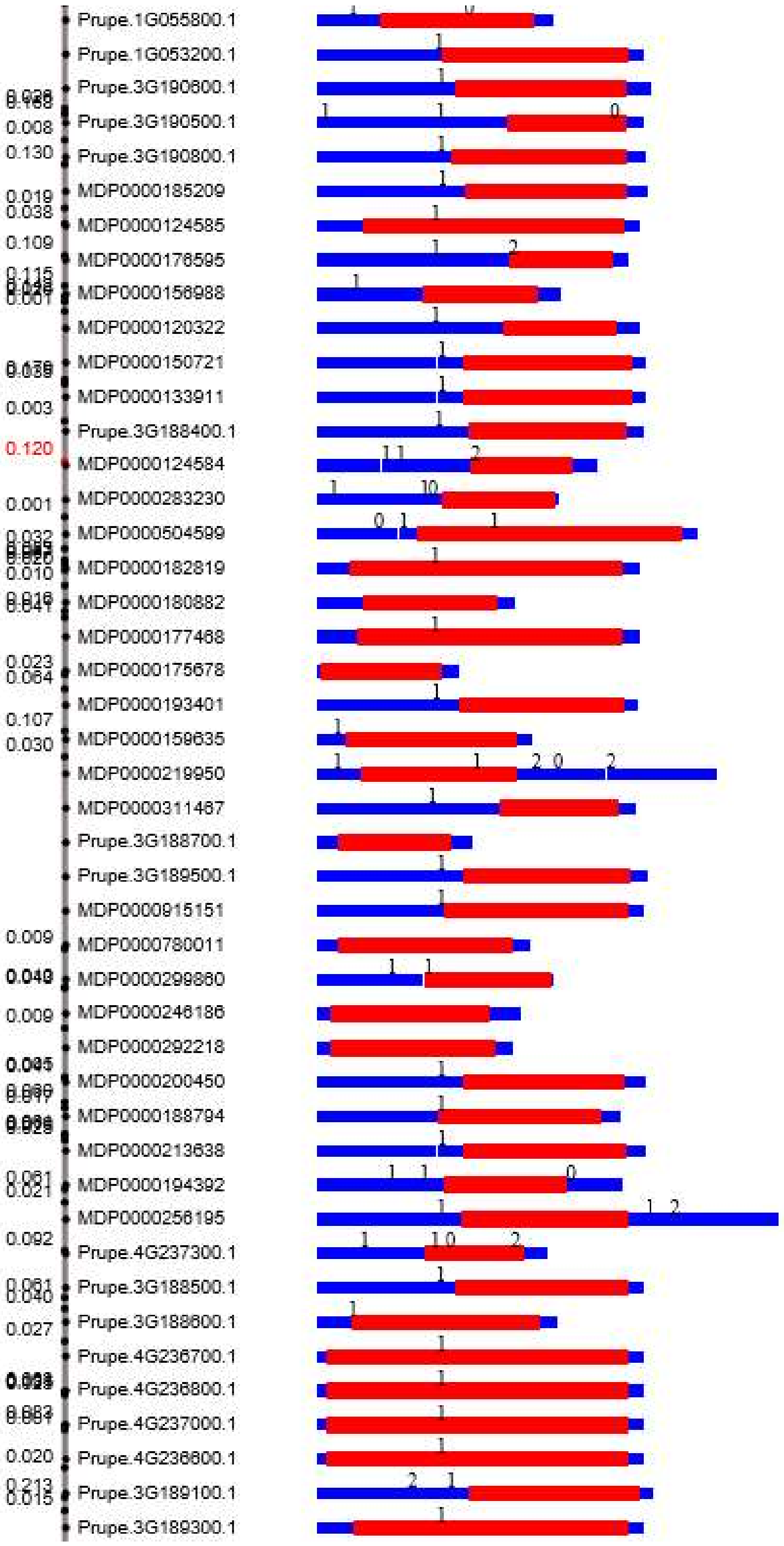

Prupe.1G055800.1
Prupe.1G053200.1
Prupe.3G190600.1
Prupe.3G190500.1
Prupe.3G190800.1
MDP0000185209
MDP0000124585
MDP0000178595
MDP0000156988
MDP0000120322
MDP0000150721
MDP0000133911
Prupe.3G188400.1
MDP0000124584
MDP0000283230
MDP0000504599
MDP0000182819
MDP0000180882
MDP0000177468
MDP0000175678
MDP0000193401
MDP0000159635
MDP0000219950
MDP0000311467
Prupe.3G188700.1
Prupe.3G189500.1
MDP0000915151
MDP0000780011
MDP0000299860
MDP0000246186
MDP0000292218
MDP0000200450
MDP0000188794
MDP0000213638
MDP0000194392
MDP0000256195
Prupe.4G237300.1
Prupe.3G188500.1
Prupe.3G188600.1
Prupe.4G236700.1
Prupe.4G236800.1
Prupe.4G237000.1
Prupe.4G236600.1
Prupe.3G189100.1
Prupe.3G189300.1

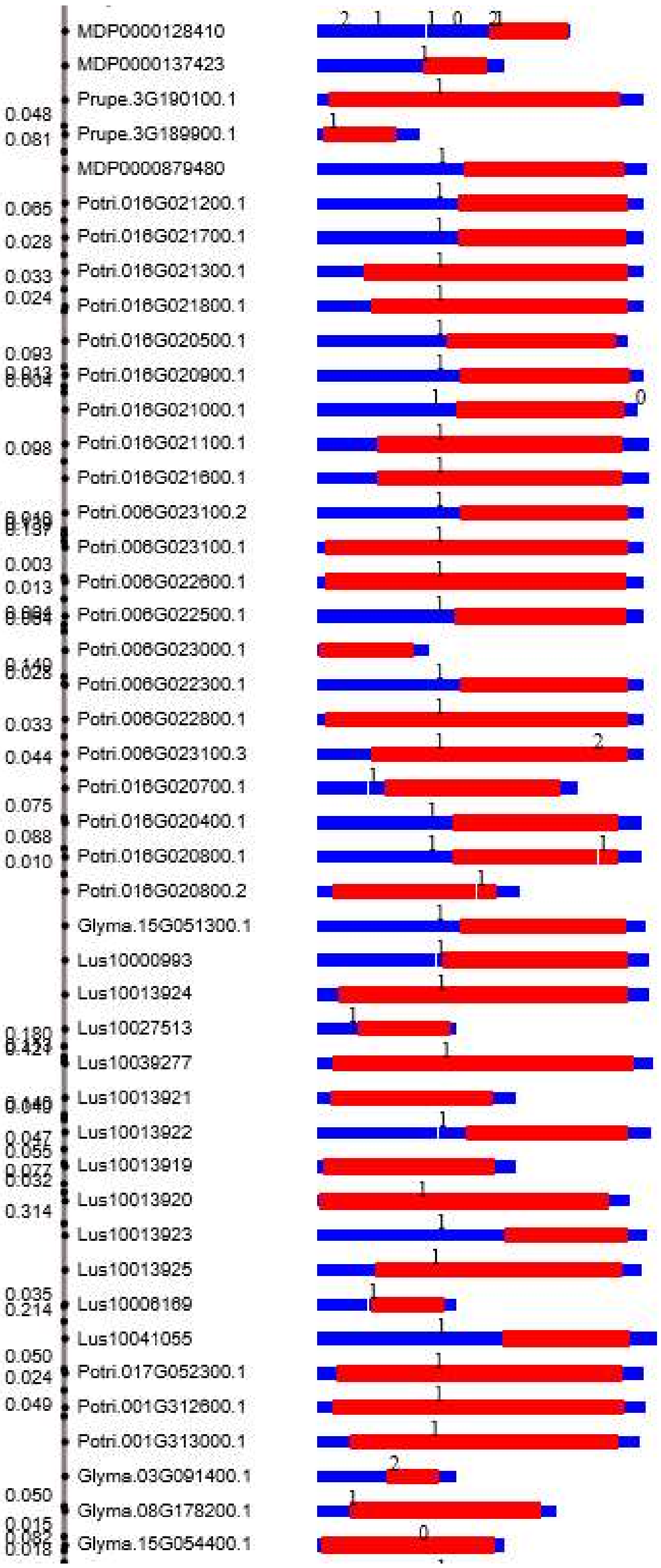

MDP0000128410
MDP0000137423
Prupe.3G190100.1
Prupe.3G189900.1
MDP0000879480
Potri.016G021200.1
Potri.016G021700.1
Potri.016G021300.1
Potri.016G021800.1
Potri.016G020500.1
Potri.016G020900.1
Potri.016G021000.1
Potri.016G021100.1
Potri.016G021600.1
Potri.006G023100.2
Potri.006G023100.1
Potri.006G022600.1
Potri.006G022500.1
Potri.006G023000.1
Potri.006G022300.1
Potri.006G022800.1
Potri.006G023100.3
Potri.016G020700.1
Potri.016G020400.1
Potri.016G020800.1
Potri.016G020800.2
Glyma.15G051300.1
Lus10000993
Lus10013924
Lus10027513
Lus10039277
Lus10013921
Lus10013922
Lus10013919
Lus10013920
Lus10013923
Lus10013925
Lus10006169
Lus10041055
Potri.017G052300.1
Potri.001G312600.1
Potri.001G313000.1
Glyma.03G091400.1
Glyma.08G178200.1
Glyma.15G054400.1
0.048
0.081
0.065
0.028
0.033
0.024
0.093
0.098
0.003
0.013
0.033
0.044
0.075
0.088
0.010
0.314
0.050
0.024
0.049
0.050

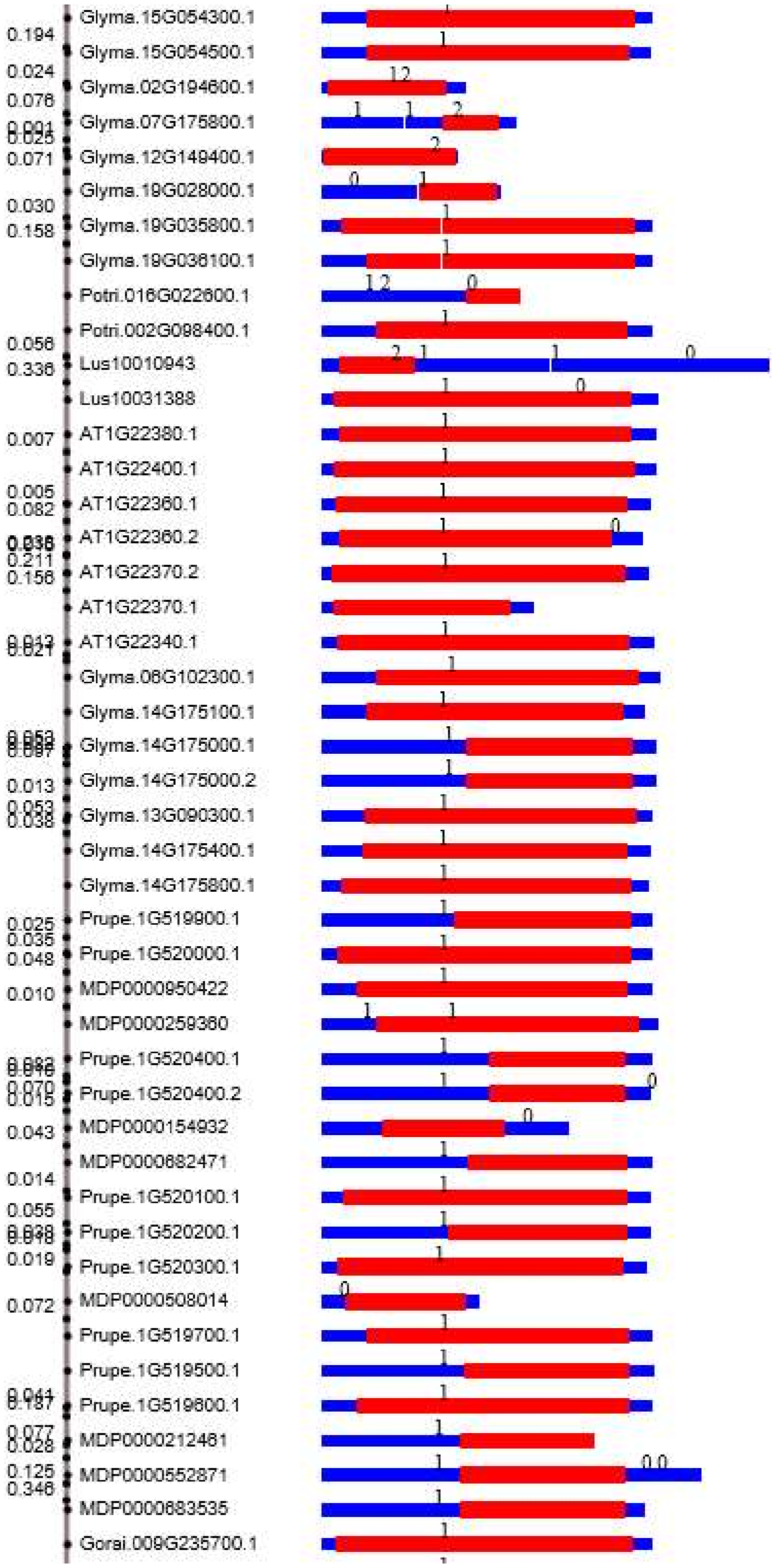

Glyma.15G054300.1
Glyma.15G054500.1
Glyma.02G194600.1
Glyma.07G175800.1
Glyma.12G149400.1
Glyma.19G028000.1
Glyma.19G035800.1
Glyma.19G036100.1
Potri.016G022600.1
Potri.002G098400.1
Lus10010943
Lus10031388
AT1G22380.1
AT1G22400.1
AT1G22360.1
AT1G22360.2
AT1G22370.2
AT1G22370.1
AT1G22340.1
Glyma.06G102300.1
Glyma.14G175100.1
Glyma.14G175000.1
Glyma.14G175000.2
Glyma.13G090300.1
Glyma.14G175400.1
Glyma.14G175800.1
Prupe.1G519900.1
Prupe.1G520000.1
MDP0000950422
MDP0000259360
Prupe.1G520400.1
Prupe.1G520400.2
MDP0000154932
MDP0000682471
Prupe.1G520100.1
Prupe.1G520200.1
Prupe.1G520300.1
MDP0000508014
Prupe.1G519700.1
Prupe.1G519500.1
Prupe.1G519600.1
MDP0000212461
MDP0000552871
MDP0000683535
Gorai.009G235700.1

Supplementary Figure S3. Gene structure comparison among eight eudicots. The intron insertion and phase 1 among 150-200 amino acids remained highly conserved during the evolution of UGT gene family in eudicots.

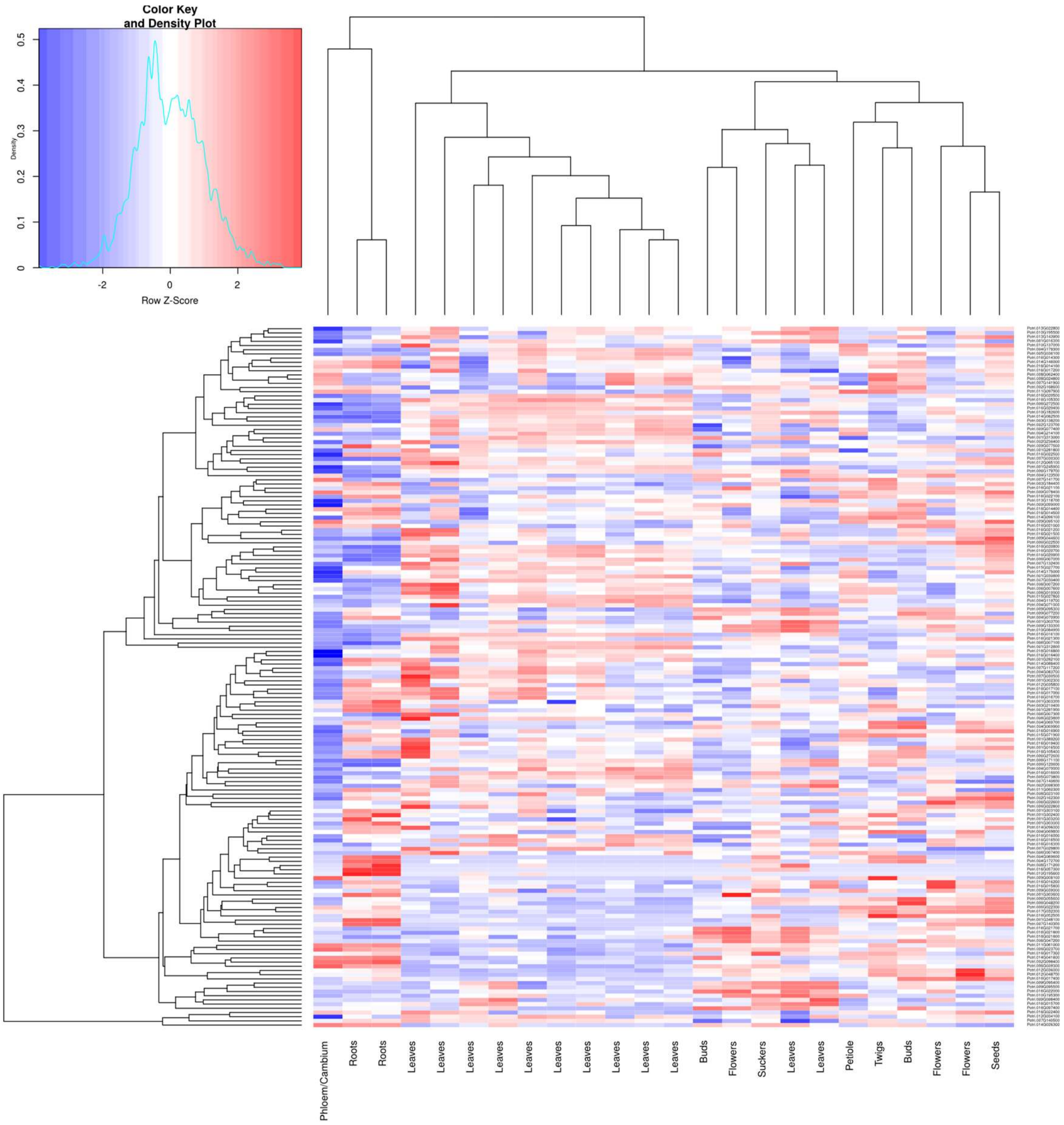

Supplementary Figure S4. Heat map of 168 UGTs expressed in various tissues in populus. Maximum expression was found in roots, leaves, and flowers.

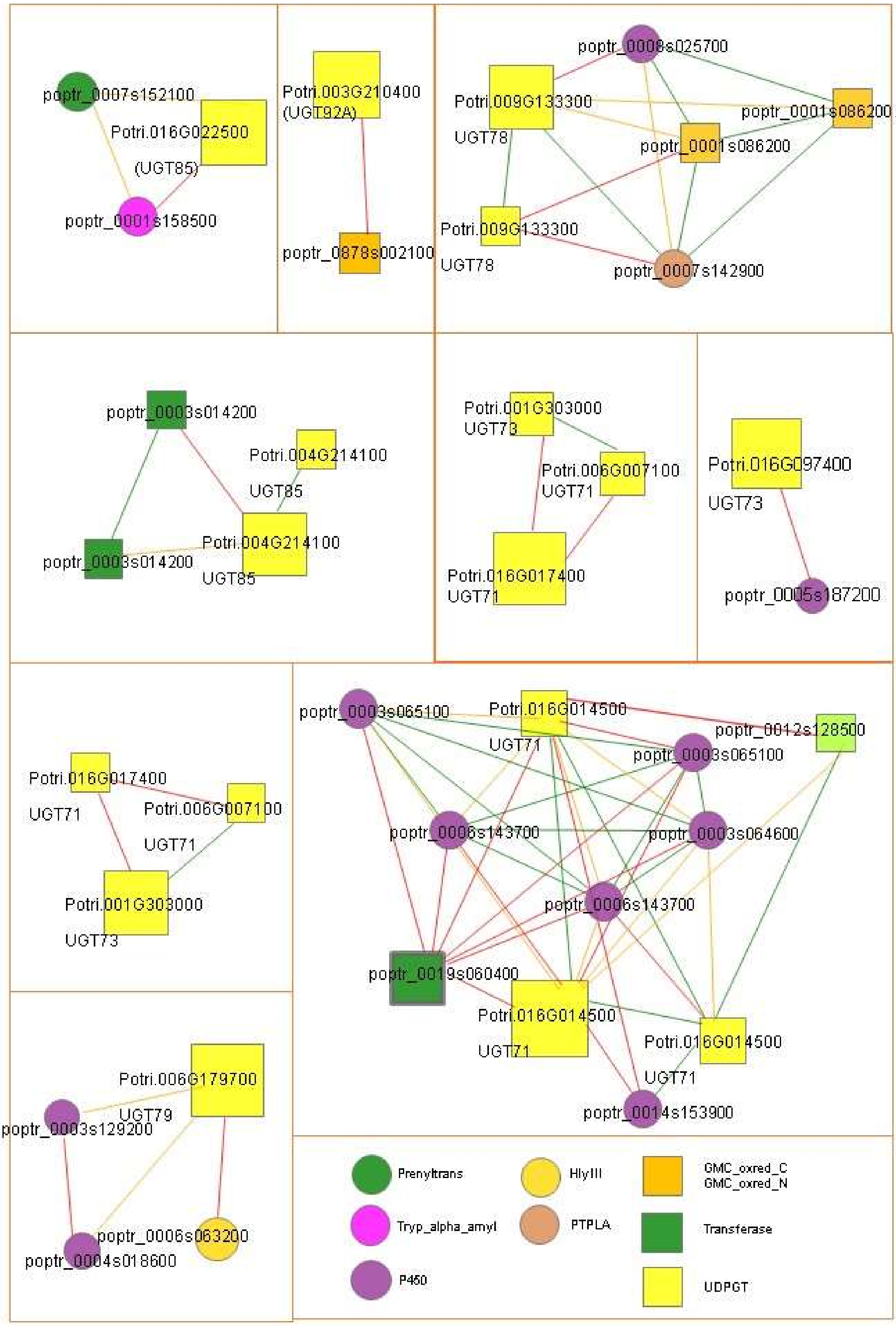
poptr_0007s152100
Potri.016G022500
(UGT85)
poptr_0001s158500
Potri.003G210400
(UGT92A)
poptr_0878s002100
poptr_0008s025700
Potri.009G133300
UGT78
poptr_0001s086200
poptr_0001s086200
Potri.009G133300
UGT78
poptr_0007s142900
poptr_0003s014200
Potri.004G214100
UGT85
poptr_0003s014200
Potri.004G214100
UGT85
Potri.001G303000
UGT73
Potri.006G007100
UGT71
Potri.016G017400
UGT71
Potri.016G097400
UGT73
poptr_0005s187200
Potri.016G017400
UGT71
Potri.006G007100
UGT71
Potri.001G303000
UGT73
poptr_0003s065100
Potri.016G014500
UGT71
poptr_0012s128500
poptr_0003s065100
poptr_0006s143700
poptr_0003s064600
poptr_0006s143700
poptr_0019s060400
Potri.016G014500
UGT71
Potri.016G014500
UGT71
poptr_0014s153900
Potri.006G179700
UGT79
poptr_0003s129200
poptr_0006s063200
poptr_0004s018600
Prenyltrans
Hly III
GMC_oxred_C
GMC_oxred_N
Tryp_alpha_amyl
PTPLA
Transferase
P450
UDPGT

Supplementary Figure S5. Co expression networking of UGT genes in Populus. First neighborhood modules were detected for co expression analysis. Yellow color boxes are showing the putative UGTs and their types while the other colors are showing their co expressed genes in first neighborhood.
